# The genome sequence of a click beetle, *Melanotus villosus *(Geoffroy in Fourcroy, 1785)

**DOI:** 10.12688/wellcomeopenres.21087.1

**Published:** 2024-03-01

**Authors:** Duncan Sivell, Maxwell V. L. Barclay, Howard Mendel, Natural History Museum Genome Acquisition Lab, Darwin Tree of Life Barcoding collective, Wellcome Sanger Institute Tree of Life Management, Samples and Laboratory team, Wellcome Sanger Institute Scientific Operations: Sequencing Operations, Wellcome Sanger Institute Tree of Life Core Informatics team, Tree of Life Core Informatics collective, Darwin Tree of Life Consortium

**Affiliations:** 1 Natural History Museum, London, England, UK

**Keywords:** Melanotus villosus, click beetle, genome sequence, chromosomal, Coleoptera

## Abstract

We present a genome assembly from an individual female *Melanotus villosus* (click beetle; Arthropoda; Insecta; Coleoptera; Elateridae). The genome sequence is 803.5 megabases in span. Most of the assembly is scaffolded into 10 chromosomal pseudomolecules, including the X sex chromosome. The mitochondrial genome has also been assembled and is 15.91 kilobases in length.

## Species taxonomy

Eukaryota; Opisthokonta; Metazoa; Eumetazoa; Bilateria; Protostomia; Ecdysozoa; Panarthropoda; Arthropoda; Mandibulata; Pancrustacea; Hexapoda; Insecta; Dicondylia; Pterygota; Neoptera; Endopterygota; Coleoptera; Polyphaga; Elateriformia; Elateroidea; Elateridae; Melanotinae; *Melanotus*; *Melanotus villosus* (Geoffroy in Fourcroy, 1785) (NCBI:txid346801).

## Background

*Melanotus villosus* is a large black click beetle, 13–20 mm long, which is associated with mature woodland where the larvae develop in rotting wood ( Duff, 2020; James, 2018; Laibner, 2000). The adult beetles are typically found on tree trunks, inside wood, in flight during the evening, or can be swept from vegetation. They can potentially be seen in all months of the year ( du Chatenet, 2000; Duff, 2020).

*Melanotus villosus* and its close relative *M. castanipes* used to be regarded as one species in Britain until they were reviewed and separated by Mendel (2004). The length of the antennae and the shape of the pronotum are useful characters for identification, though separation of adults is difficult, and larval characters may also be helpful (Mendel, 2004). Pre-2004 records of *M. villosus* may therefore refer to *M. castanipes* and should be treated with caution. The names *Melanotus rufipes* (Herbst, 1784) non (De Geer, 1774) and *M. erythropus* (Gmelin, 1790), now treated as synonyms of *M. villosus*, were frequently applied to both species in the older British literature (Duff, 2018).

It appears that *M. villosus* mostly occurs in the south-east of England while *M. castanipes* is widespread across Britain ( Duff, 2020; Mendel, 2004). On mainland Europe this situation is reversed, with *M. villosus* being the more widespread species while *M. castanipes* tends to occur at higher elevations ( du Chatenet, 2000; Laibner, 2000).

There are some ecological differences between these species that are worth noting. James (2018) reports that in Hertfordshire *M. villosus* is closely associated with woodland, while *M. castanipes* tends to occur in more open habitats (while still requiring dead wood). He states that there is only one site in Hertfordshire where both beetles are known to coexist. Some authors have suggested that in continental Europe, *M. villosus* adults are diurnal whereas *M. castanipes* are crepuscular or nocturnal and sometimes come to light traps ( Laibner, 2000; Leseigneur, 1972). Such differences have not yet been observed in Britain. The global distributions of these species differ, as *M. villosus* is Palaearctic whereas *M. castanipes* is Holarctic, also occurring in North America ( Laibner, 2000).

The genome of *Melanotus villosus* was sequenced as part of the Darwin Tree of Life Project, a collaborative effort to sequence all named eukaryotic species in the Atlantic Archipelago of Britain and Ireland. Here we present a chromosomally complete genome sequence based on one male specimen (NHMUK014111204) collected by DS on 20/04/2021 from Bookham Commons and identified by MB and HM. This genome note will provide a molecular description of this species and will be useful for separating *M. villosus* from its close relative *M. castanipes* in future research projects.

## Genome sequence report

**Figure 1.**
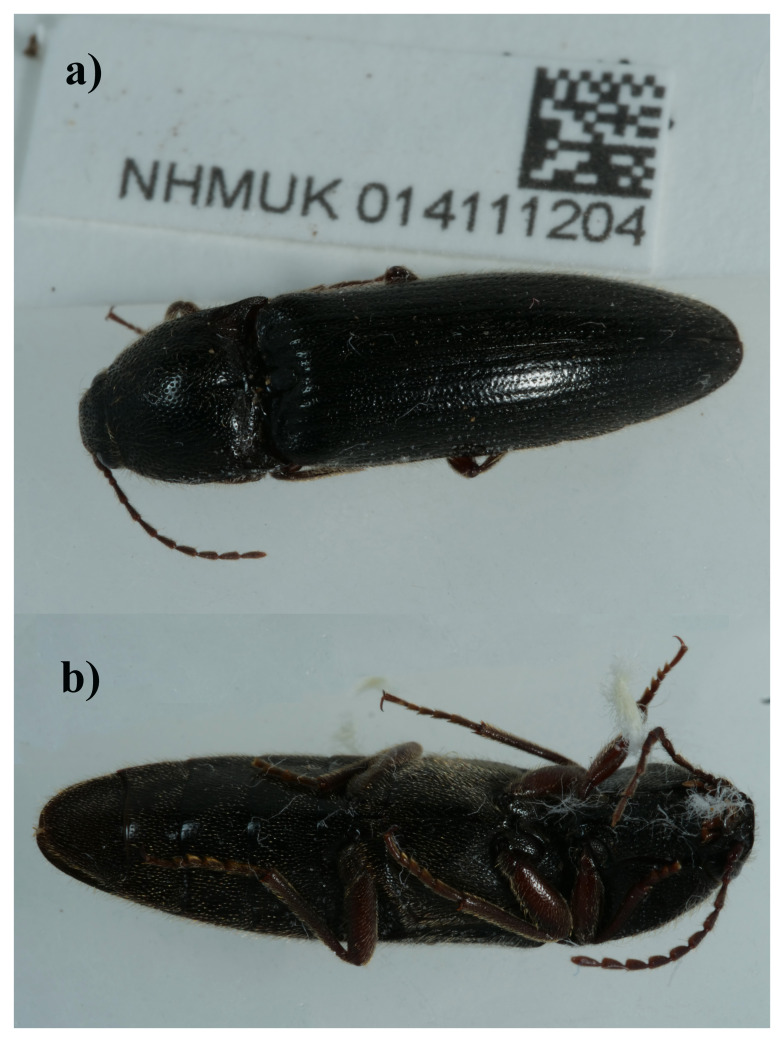
Photograph of the *Melanotus villosus* (icMelVill1) specimen used for genome sequencing. **a**) Dorsal view, **b**) Ventral view.

The genome was sequenced from one female *Melanotus villosus* ( Figure 1) collected from Bookham Commons, England, UK (51.29, –0.39). A total of 29-fold coverage in Pacific Biosciences single-molecule HiFi long reads was generated. Primary assembly contigs were scaffolded with chromosome conformation Hi-C data. Manual assembly curation corrected 30 missing joins or mis-joins and removed 5 haplotypic duplications, reducing the assembly length by 0.91% and the scaffold number by 20.20%, and increasing the scaffold N50 by 28.53%.

**Figure 2.**
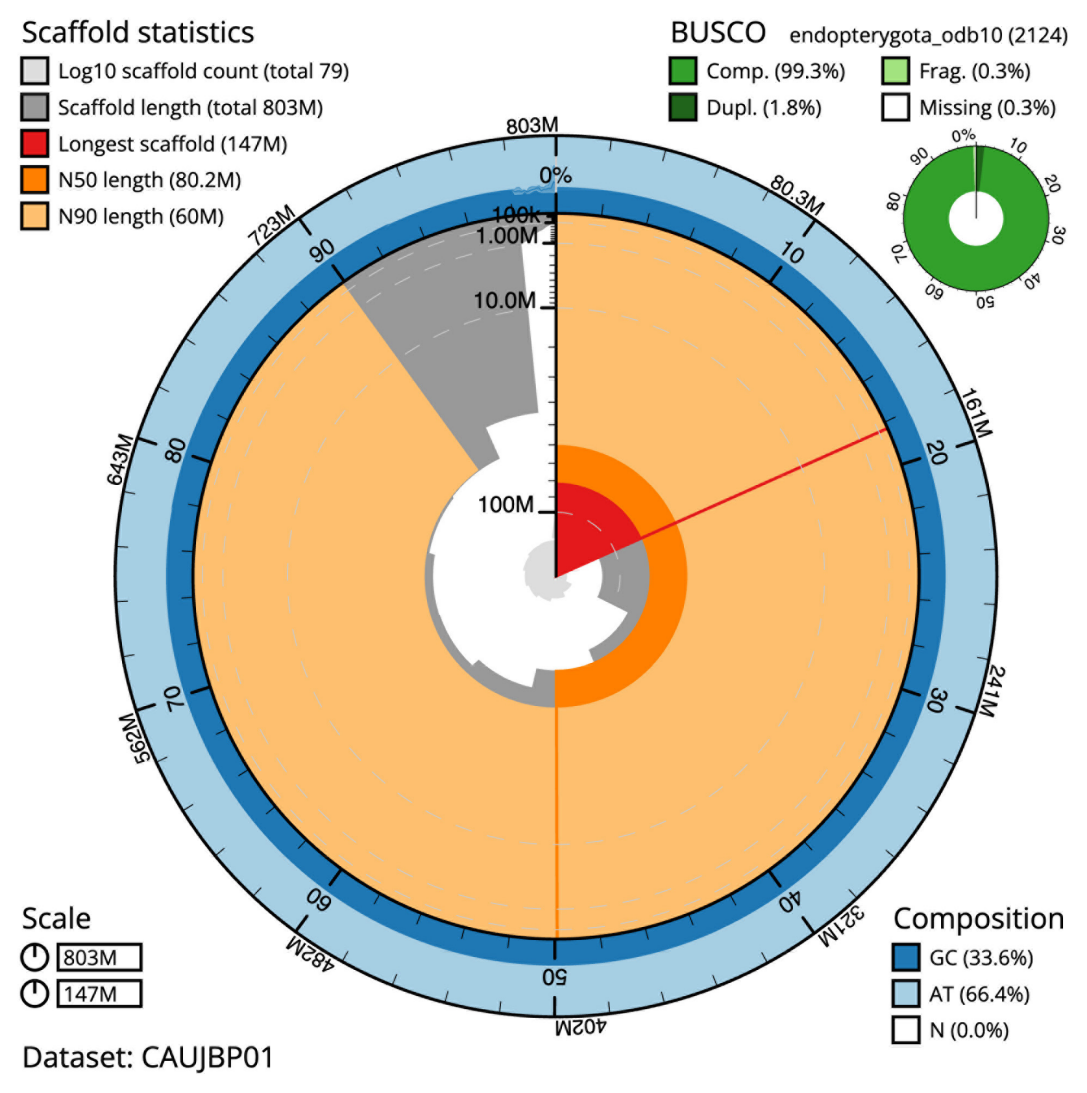
Genome assembly of *Melanotus villosus*, icMelVill1.1: metrics. The BlobToolKit snail plot shows N50 metrics and BUSCO gene completeness. The main plot is divided into 1,000 size-ordered bins around the circumference with each bin representing 0.1% of the 803,481,935 bp assembly. The distribution of scaffold lengths is shown in dark grey with the plot radius scaled to the longest scaffold present in the assembly (147,383,313 bp, shown in red). Orange and pale-orange arcs show the N50 and N90 scaffold lengths (80,210,941 and 59,994,649 bp), respectively. The pale grey spiral shows the cumulative scaffold count on a log scale with white scale lines showing successive orders of magnitude. The blue and pale-blue area around the outside of the plot shows the distribution of GC, AT and N percentages in the same bins as the inner plot. A summary of complete, fragmented, duplicated and missing BUSCO genes in the endopterygota_odb10 set is shown in the top right. An interactive version of this figure is available at https://blobtoolkit.genomehubs.org/view/CAUJBP01/dataset/CAUJBP01/snail.

**Figure 3.**
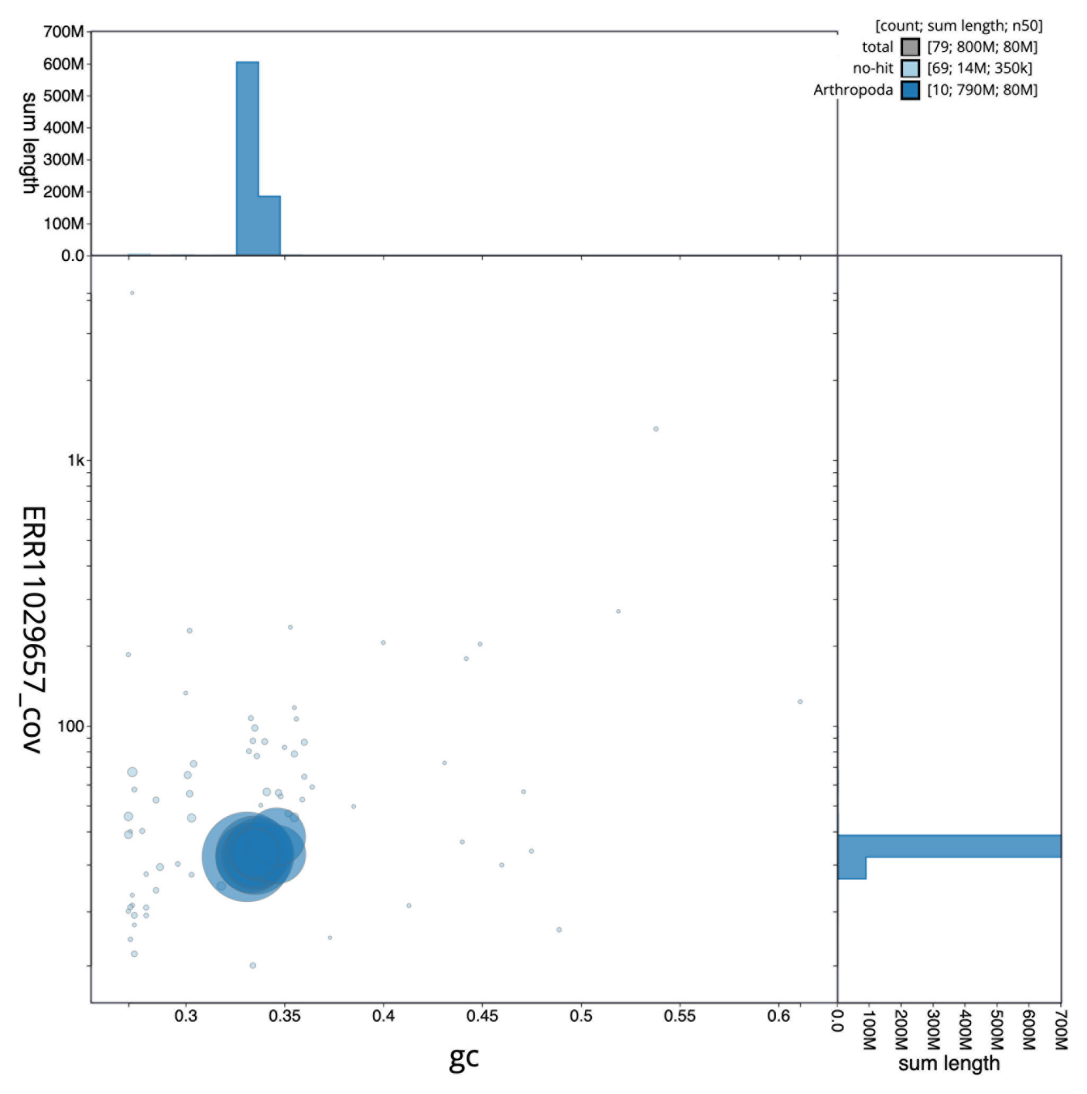
Genome assembly of *Melanotus villosus*, icMelVill1.1: BlobToolKit GC-coverage plot. Sequences are coloured by phylum. Circles are sized in proportion to sequence length. Histograms show the distribution of sequence length sum along each axis. An interactive version of this figure is available at https://blobtoolkit.genomehubs.org/view/CAUJBP01/dataset/CAUJBP01/blob.

**Figure 4.**
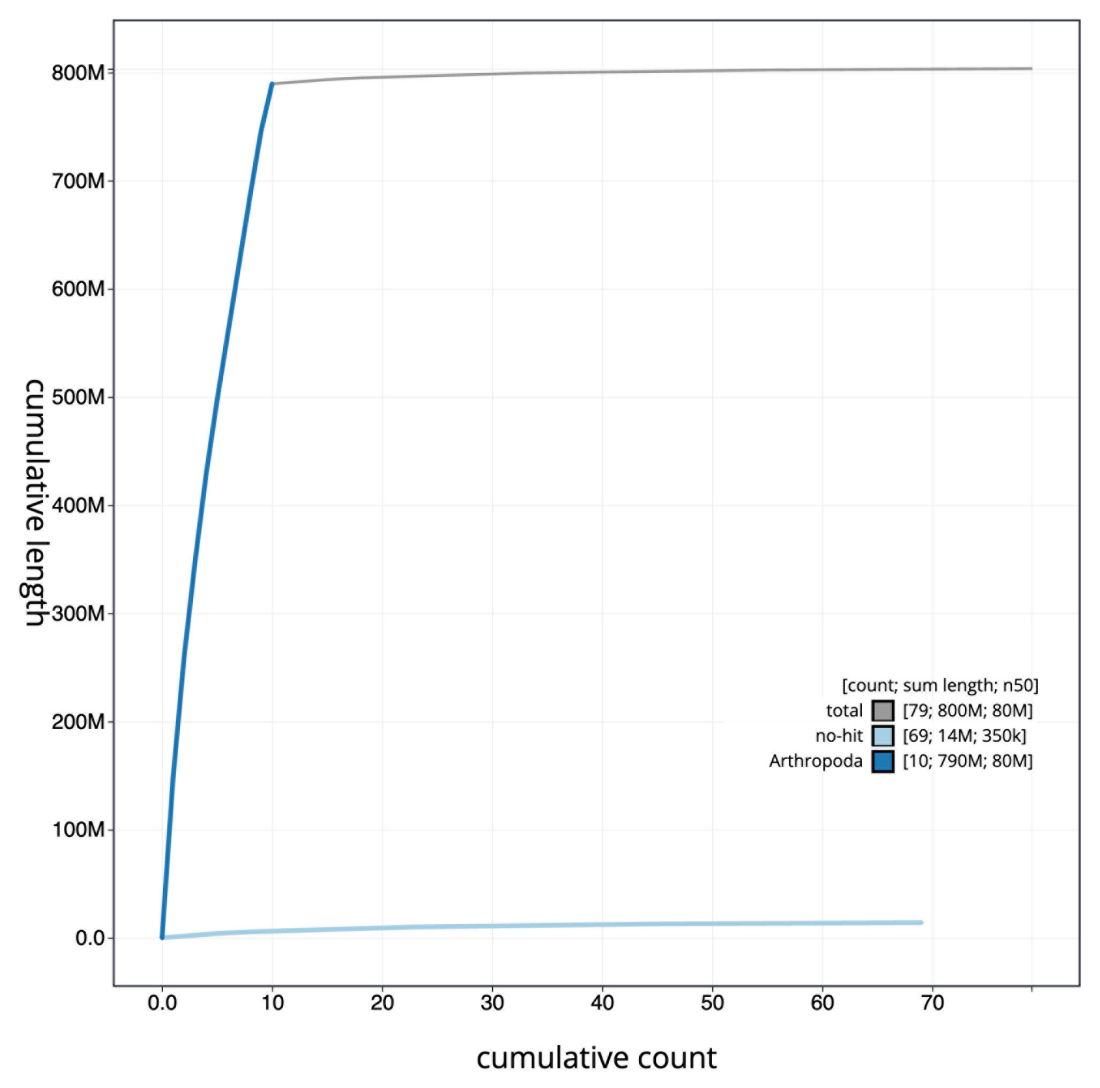
Genome assembly of *Melanotus villosus*, icMelVill1.1: BlobToolKit cumulative sequence plot. The grey line shows cumulative length for all sequences. Coloured lines show cumulative lengths of sequences assigned to each phylum using the buscogenes taxrule. An interactive version of this figure is available at https://blobtoolkit.genomehubs.org/view/CAUJBP01/dataset/CAUJBP01/cumulative.

**Figure 5.**
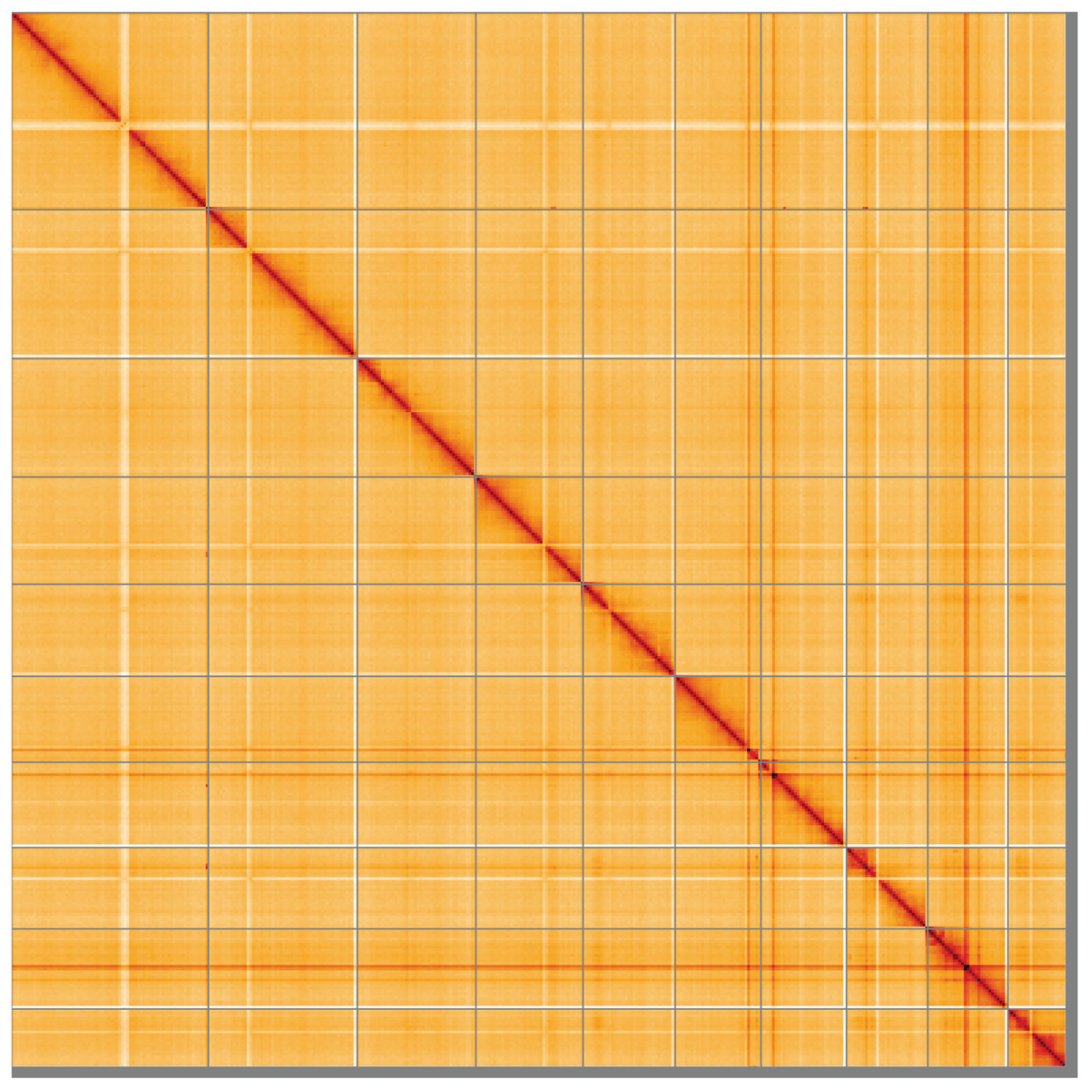
Genome assembly of *Melanotus villosus*, icMelVill1.1: Hi-C contact map of the icMelVill1.1 assembly, visualised using HiGlass. Chromosomes are shown in order of size from left to right and top to bottom. An interactive version of this figure may be viewed at https://genome-note-higlass.tol.sanger.ac.uk/l/?d=YzPOQ6dBT_qs6yvQPYKXWA.

The final assembly has a total length of 803.5 Mb in 78 sequence scaffolds with a scaffold N50 of 80.2 Mb ( Table 1). The snail plot in Figure 2 provides a summary of the assembly statistics, while the distribution of assembly scaffolds on GC proportion and coverage is shown in Figure 3. The cumulative assembly plot in Figure 4 shows curves for subsets of scaffolds assigned to different phyla. Most (98.25%) of the assembly sequence was assigned to 10 chromosomal-level scaffolds, representing 9 autosomes and the X sex chromosome. Chromosome-scale scaffolds confirmed by the Hi-C data are named in order of size ( Figure 5; Table 2). Chromosome X was assigned by alignment to the genome assembly for *Agrypnus murinus* (GCA_929113105.1) ( Crowley *et al.*, 2023). While not fully phased, the assembly deposited is of one haplotype. Contigs corresponding to the second haplotype have also been deposited. The mitochondrial genome was also assembled and can be found as a contig within the multifasta file of the genome submission.

**Table 1.** Genome data for *Melanotus villosus*, icMelVill1.1.

Project accession data		
Assembly identifier	icMelVill1.1	
Species	*Melanotus villosus*	
Specimen	icMelVill1	
NCBI taxonomy ID	346801	
BioProject	PRJEB60633	
BioSample ID	SAMEA9359409	
Isolate information	icMelVill1 icMelVill1	
Assembly metrics *		*Benchmark*
Consensus quality (QV)	67.2	*≥ 50*
*k*-mer completeness	100.0%	*≥ 95%*
BUSCO **	C:99.3%[S:97.5%,D:1.8%], F:0.3%,M:0.3%,n:2,124	*C ≥ 95%*
Percentage of assembly mapped to chromosomes	98.25%	*≥ 95%*
Sex chromosomes	X	*localised homologous pairs*
Organelles	Mitochondrial genome: 15.91 kb	*complete single alleles*
Raw data accessions		
PacificBiosciences SEQUEL II	ERR11029657	
Hi-C Illumina	ERR11040161	
Genome assembly		
Assembly accession	GCA_963082815.1	
*Accession of alternate haplotype*	GCA_963082845.1	
Span (Mb)	803.5	
Number of contigs	187	
Contig N50 length (Mb)	13.8	
Number of scaffolds	78	
Scaffold N50 length (Mb)	80.2	
Longest scaffold (Mb)	147.38	

* Assembly metric benchmarks are adapted from column VGP-2020 of “Table 1: Proposed standards and metrics for defining genome assembly quality” from Rhie *et al.* (2021). ** BUSCO scores based on the endopterygota_odb10 BUSCO set using version 5.3.2. C = complete [S = single copy, D = duplicated], F = fragmented, M = missing, n = number of orthologues in comparison. A full set of BUSCO scores is available at https://blobtoolkit.genomehubs.org/view/CAUJBP01/dataset/CAUJBP01/busco.

**Table 2.** Chromosomal pseudomolecules in the genome assembly of *Melanotus villosus*, icMelVill1.

INSDC accession	Chromosome	Length (Mb)	GC%
OY720418.1	1	147.38	33.0
OY720419.1	2	111.47	33.5
OY720420.1	3	88.75	33.5
OY720421.1	4	80.21	33.5
OY720422.1	5	68.93	33.5
OY720423.1	6	64.26	33.5
OY720424.1	7	63.92	34.0
OY720425.1	8	60.85	34.5
OY720426.1	9	59.99	34.5
OY720427.1	X	43.66	33.5
OY720428.1	MT	0.02	27.5

The estimated Quality Value (QV) of the final assembly is 67.2 with *k*-mer completeness of 100.0%, and the assembly has a BUSCO v5.3.2 completeness of 99.3% (single = 97.5%, duplicated = 1.8%), using the endopterygota_odb10 reference set ( *n* = 2,124).

Metadata for specimens, barcode results, spectra estimates, sequencing runs, contaminants and pre-curation assembly statistics are given at https://links.tol.sanger.ac.uk/species/346801.

## Methods

### Sample acquisition and nucleic acid extraction

A female *Melanotus villosus* (specimen ID NHMUK014111204, ToLID icMelVill1) was hand-picked in Bookham Commons, England, UK (latitude 51.29, longitude –0.39) on 2021-04-20. The specimen was collected by Duncan Sivell (Natural History Museum) and identified by Maxwell Barclay and Howard Mendel (Natural History Museum) and preserved by dry freezing at – 80°C.

The workflow for high molecular weight (HMW) DNA extraction at the Wellcome Sanger Institute (WSI) includes a sequence of core procedures: sample preparation; sample homogenisation, DNA extraction, fragmentation, and clean-up. In sample preparation, the icMelVill1 sample was weighed and dissected on dry ice ( Jay *et al.*, 2023). Tissue from the abdomen was homogenised using a PowerMasher II tissue disruptor ( Denton *et al.*, 2023a).

HMW DNA was extracted using the Automated MagAttract v1 protocol ( Sheerin *et al.*, 2023). DNA was sheared into an average fragment size of 12–20 kb in a Megaruptor 3 system ( Todorovic *et al.*, 2023). Sheared DNA was purified by solid-phase reversible immobilisation ( Strickland *et al.*, 2023): in brief, the method employs a 1.8X ratio of AMPure PB beads to sample to eliminate shorter fragments and concentrate the DNA. The concentration of the sheared and purified DNA was assessed using a Nanodrop spectrophotometer and Qubit Fluorometer and Qubit dsDNA High Sensitivity Assay kit. Fragment size distribution was evaluated by running the sample on the FemtoPulse system.

Protocols developed by the WSI Tree of Life laboratory are publicly available on protocols.io ( Denton *et al.*, 2023b).

### Sequencing

Pacific Biosciences HiFi circular consensus DNA sequencing libraries were constructed according to the manufacturers’ instructions. DNA sequencing was performed by the Scientific Operations core at the WSI on a Pacific Biosciences SEQUEL II instrument. Hi-C data were also generated from head tissue of icMelVill1 using the Arima2 kit and sequenced on the Illumina NovaSeq 6000 instrument.

### Genome assembly, curation and evaluation

Assembly was carried out with Hifiasm ( Cheng *et al.*, 2021) and haplotypic duplication was identified and removed with purge_dups ( Guan *et al.*, 2020). The assembly was then scaffolded with Hi-C data ( Rao *et al.*, 2014) using YaHS ( Zhou *et al.*, 2023). The assembly was checked for contamination and corrected as described previously ( Howe *et al.*, 2021). Manual curation was performed using HiGlass ( Kerpedjiev *et al.*, 2018) and PretextView ( Harry, 2022). The mitochondrial genome was assembled using MitoHiFi ( Uliano-Silva *et al.*, 2023), which runs MitoFinder ( Allio *et al.*, 2020) or MITOS ( Bernt *et al.*, 2013) and uses these annotations to select the final mitochondrial contig and to ensure the general quality of the sequence.

A Hi-C map for the final assembly was produced using bwa-mem2 ( Vasimuddin *et al.*, 2019) in the Cooler file format ( Abdennur & Mirny, 2020). To assess the assembly metrics, the *k*-mer completeness and QV consensus quality values were calculated in Merqury ( Rhie *et al.*, 2020). This work was done using Nextflow ( Di Tommaso *et al.*, 2017) DSL2 pipelines “sanger-tol/readmapping” ( Surana *et al.*, 2023a) and “sanger-tol/genomenote” ( Surana *et al.*, 2023b). The genome was analysed within the BlobToolKit environment ( Challis *et al.*, 2020) and BUSCO scores ( Manni *et al.*, 2021; Simão *et al.*, 2015) were calculated.

Table 3 contains a list of relevant software tool versions and sources.

**Table 3.** Software tools: versions and sources.

Software tool	Version	Source
BlobToolKit	4.2.1	https://github.com/blobtoolkit/blobtoolkit
BUSCO	5.3.2	https://gitlab.com/ezlab/busco
Hifiasm	0.16.1-r375	https://github.com/chhylp123/hifiasm
HiGlass	1.11.6	https://github.com/higlass/higlass
Merqury	MerquryFK	https://github.com/thegenemyers/MERQURY.FK
MitoHiFi	2	https://github.com/marcelauliano/MitoHiFi
PretextView	0.2	https://github.com/wtsi-hpag/PretextView
purge_dups	1.2.3	https://github.com/dfguan/purge_dups
sanger-tol/genomenote	v1.0	https://github.com/sanger-tol/genomenote
sanger-tol/readmapping	1.1.0	https://github.com/sanger-tol/readmapping/tree/1.1.0
YaHS	1.2a	https://github.com/c-zhou/yahs

### Wellcome Sanger Institute – Legal and Governance

The materials that have contributed to this genome note have been supplied by a Darwin Tree of Life Partner. The submission of materials by a Darwin Tree of Life Partner is subject to the **‘Darwin Tree of Life Project Sampling Code of Practice’**, which can be found in full on the Darwin Tree of Life website here. By agreeing with and signing up to the Sampling Code of Practice, the Darwin Tree of Life Partner agrees they will meet the legal and ethical requirements and standards set out within this document in respect of all samples acquired for, and supplied to, the Darwin Tree of Life Project.

Further, the Wellcome Sanger Institute employs a process whereby due diligence is carried out proportionate to the nature of the materials themselves, and the circumstances under which they have been/are to be collected and provided for use. The purpose of this is to address and mitigate any potential legal and/or ethical implications of receipt and use of the materials as part of the research project, and to ensure that in doing so we align with best practice wherever possible. The overarching areas of consideration are:

•   Ethical review of provenance and sourcing of the material

•   Legality of collection, transfer and use (national and international) 

Each transfer of samples is further undertaken according to a Research Collaboration Agreement or Material Transfer Agreement entered into by the Darwin Tree of Life Partner, Genome Research Limited (operating as the Wellcome Sanger Institute), and in some circumstances other Darwin Tree of Life collaborators.

## Data Availability

European Nucleotide Archive: *Melanotus villosus*. Accession number PRJEB60633; https://identifiers.org/ena.embl/PRJEB60633 ( Wellcome Sanger Institute, 2023). The genome sequence is released openly for reuse. The *Melanotus villosus* genome sequencing initiative is part of the Darwin Tree of Life (DToL) project. All raw sequence data and the assembly have been deposited in INSDC databases. The genome will be annotated using available RNA-Seq data and presented through the Ensembl pipeline at the European Bioinformatics Institute. Raw data and assembly accession identifiers are reported in Table 1.

## Author information

Members of the Natural History Museum Genome Acquisition Lab are listed here: https://doi.org/10.5281/zenodo.7139035.

Members of the Darwin Tree of Life Barcoding collective are listed here: https://doi.org/10.5281/zenodo.4893703.

Members of the Wellcome Sanger Institute Tree of Life Management, Samples and Laboratory team are listed here: https://doi.org/10.5281/zenodo.10066175.

Members of Wellcome Sanger Institute Scientific Operations: Sequencing Operations are listed here: https://doi.org/10.5281/zenodo.10043364.

Members of the Wellcome Sanger Institute Tree of Life Core Informatics team are listed here: https://doi.org/10.5281/zenodo.10066637.

Members of the Tree of Life Core Informatics collective are listed here: https://doi.org/10.5281/zenodo.5013541.

Members of the Darwin Tree of Life Consortium are listed here: https://doi.org/10.5281/zenodo.4783558.
