# Supplementary material for: The genome sequence of a click beetle, Melanotus villosus (Geoffroy in Fourcroy, 1785)
Source: Wellcome Open Res. 2024 Mar 1;9:108. [Version 1] doi: 10.12688/wellcomeopenres.21087.1 (PMC11347919; doi:10.12688/wellcomeopenres.21087.1)
Supplement: Peer review history

# Reviewer report: Reviewer response for version 1

**DOI:** 10.21956/wellcomeopenres.23327.r89162

**Published:** 2024-08-26

**Authors:** Luciano Palmieri

**Affiliations:** 1 Free University of Bolzen-Bolzano, Bolzano, Italy; 2 University of Wisconsin-Madison, Madison, WI, USA

The study presents a genome assembly of a female *Melanotus villosus*, a click beetle (Coleoptera; Elateridae). The genome sequence spans 803.5 Mb, scaffolded into 10 chromosomal pseudomolecules, including the X sex chromosome. The mitochondrial genome is also assembled, measuring 15.91 kb. This work clarifies the taxonomy of *M. villosus* and its ecological significance, providing a valuable resource for future research. Protocols followed best practices in genomics. The integration of PacBio HiFi long reads, Hi-C scaffolding, and quality checks ensures a high-quality assembly. Data deposition in public repositories and detailed methodological descriptions enhance reproducibility. Gene annotation is not available, but future annotation is planned. Overall, it is a valuable resource for research.

Are sufficient details of methods and materials provided to allow replication by others?

Yes

Is the rationale for creating the dataset(s) clearly described?

Yes

Are the datasets clearly presented in a useable and accessible format?

Yes

Are the protocols appropriate and is the work technically sound?

Yes

Reviewer Expertise:

Entomology, genomics.

I confirm that I have read this submission and believe that I have an appropriate level of expertise to confirm that it is of an acceptable scientific standard.

# Reviewer report: Reviewer response for version 1

**DOI:** 10.21956/wellcomeopenres.23327.r90153

**Published:** 2024-08-06

**Authors:** Zachary P Cohen

**Affiliations:** 1 USDA Agricultural Research Service, College Station, TX, USA

The genome report of a click beetle endemic to Britain presented here is well written and provides the necessary details for assembly reproduction. The authors describe the relevance of this specimen in an ecological and historical context, *ie *the past 'lumping' of this species with another click-beetle, the methods used to generate the assembly, and its importance for the larger Darwin Tree of Life Project goal to sequence all eukaryotes of Britain and Ireland. The figures and results provided are informative and help emphasize the high-quality product. This genome assembly has relevant applications for coleopterists, taxonomists and evolutionary biologists.

Are sufficient details of methods and materials provided to allow replication by others?

Yes

Is the rationale for creating the dataset(s) clearly described?

Yes

Are the datasets clearly presented in a useable and accessible format?

Yes

Are the protocols appropriate and is the work technically sound?

Yes

Reviewer Expertise:

Entomology, genome biology, population genetics.

I confirm that I have read this submission and believe that I have an appropriate level of expertise to confirm that it is of an acceptable scientific standard.
